# Escaping ageing through Cell Annealing—a phenomenological model

**DOI:** 10.1038/s41422-025-01138-z

**Published:** 2025-07-08

**Authors:** Sebastian Memczak, Juan Carlos Izpisua Belmonte, Thore Graepel

**Affiliations:** 1 Altos Labs San Diego, Institute of Science, San Diego, USA; 2 Altos Labs Cambridge, Institute of Computation, Cambridge, UK

**Keywords:** Developmental biology, Ageing

Cellular rejuvenation shows great promise for treating age-related diseases and disabilities. However, the underlying molecular mechanisms and how and where information for youthful, healthy cells might be stored remain poorly understood. This is largely due to the complexity of ageing which involves numerous molecular modalities, their interactions, and a wide array of phenotypes, making it challenging to model or even conceptualise these processes.^1^ Here, we introduce “Cell Annealing”, a phenomenological model that builds on the Waddington Landscape and features of Hopfield Networks. It provides a novel perspective on ageing, aims to deepen our understanding of cell state information storage and retrieval, and offers a framework for cell rejuvenation and therapeutic interventions.

## A cell state landscape defined by distributed information

Advances in single cell technologies have led to fine-grained descriptions of the > 200 cell types of a human body, their subtypes, and, importantly, diverse cellular states.^2,3^ These states can be thought of as regions within a continuum of a high-dimensional manifold (reviewed in ref. ^4^). This perspective aligns with Waddington’s epigenetic landscape, which likens development to a 3D topographic map.^5^ Here, a cell’s differentiation potential is analogous to altitude: pluripotent stem cells reside on mountain tops and ridges, from which they can descend along pre-defined paths into valleys representing the epigenetic configurations of fully differentiated cell types.

**Fig. 1:**
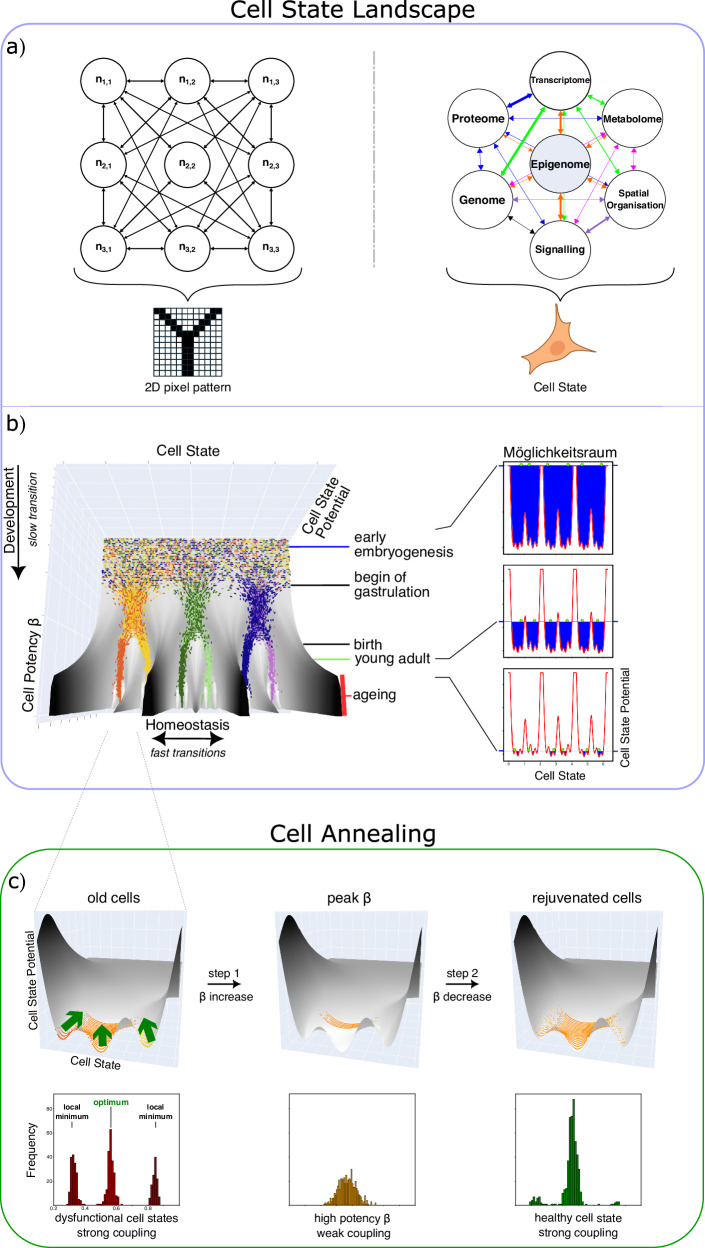
Illustration of how distributed information gives rise to a Cell State Landscape enabling rejuvenation through Cell Annealing. **a** In a Hopfield network, neurons are symmetrically connected, forming an energy landscape that stores patterns (e.g., the letter “Y”). Similarly, we model Cell States as emergent from asymmetrically interacting biological modalities. **b** *x*-axis: Cell States; *y*-axis: Cell State Potential (analogous to potential energy, drives dynamics of Cell State); *z*-axis: Cellular Potency β, determines the horizon of Cell States. Cells undergo slow transitions as Cell Potency β decreases in development and ageing. At fixed β, in homeostasis, cells shift rapidly and stochastically under the restorative force of the Cell State Potential. Colours indicate cell types, ageing regime marked by red bar. This 3D illustration serves as a representation of the underlying high-dimensional Cell State Landscape. right: high β allows access to all Cell States, the “Möglichkeitsraum” is maximal, cells are pluripotent; as β declines, the Möglichkeitsraum gets smaller and cells get trapped in suboptimal local minima, like ridges and valleys revealed by receding water. Cells in green. **c** Cell Annealing, i.e. temporarily increasing β (green arrows), expands the set of reachable cell states and enables escape from suboptimal local minima. When β is reduced again cells assume primarily healthy Cell States. Histograms show shifts in Cell State distributions at minimal potency. **b**, **c** Simulation plots for illustration purpose only.

We expand upon the landscape metaphor to model the full spectrum of cell types and states throughout a cell’s life. In our conception, a high-dimensional energy Cell State Landscape captures all molecular modalities that physically constitute a cell (Fig. 1). We model development, aging, and reprogramming as an abstract dynamical system propelled by stochastic descent along this manifold. Troughs in the landscape represent stable states surrounded by basins of attraction, where, irrespective of the initial conditions, the dynamics inevitably converge at the deepest accessible point. Once at a minimum, the cell reaches a stable state, subject only to minor fluctuations from stress and stochastic noise.

In this landscape, the *x*-axis represents the Cell State spectrum, the *y*-axis represents an abstract Cell State Potential, akin to potential energy, and the *z*-axis denotes Cell Potency β, a measure that determines which slice of Cell States are accessible to a given cell. Cell potency is highest for undifferentiated stem cells and declines with commitment to cell types and, importantly, further during ageing.^6,7^ In this model Cells States can be thought of as being ruled by the Gibbs measure, where low energy states are more probable but as potency increases higher energy states become attainable too.

We think of the Cell State Landscape as a static outcome of a species’ evolution, encompassing all viable cell states. It is shaped by the sum of the configurations of cellular modalities such as the transcriptome, proteome, interactome or spatial organization.

We envision the connectivity between state variables through an analogy to Hopfield Networks, a type of recurrent neural network that encodes information in the weights of neuronal connections that, was recently used to model enhancer-transcription factor interactions.^8,9^ In our conception each computational unit symbolizes a modality with a specific configuration at a specific time (Fig. 1). We include a few exemplary modalities that serve as high-level illustrations. For instance, we consider the signalling modality abstractly as the sum of internal and external inputs such as those from the extra cellular matrix or the endocrine system. The states of modalities interact with one another, and the strengths of interactions are characterized by different weights. For instance, the epigenome exerts strong influence over the transcriptome, whereas the reverse direction is weaker. In contrast to classical Hopfield Networks the connections here are asymmetric. Different stable network states emerge through mutual reinforcement and collectively define the landscape’s shape (Fig. 1). Note that in this model, analogous to auto-associative Hopfield Networks, any stored pattern, i.e. Cell State, can be retrieved by providing only partial and noisy information as input.

## Cell dynamics in the cell state landscape

As cells differentiate from the zygote, they are guided into one of three main valleys — ectoderm, mesoderm, or endoderm — eventually settling into smaller troughs that represent mature cell types. We propose to think of these transitions as driven by a gradual, time-dependent decline of an abstract parameter: Cell Potency β. At any given β a cell essentially behaves like a stochastic marble meandering downhill along the Cell State axis under the influence of the Cell State Potential until it settles in a trough. Therefore, two cells at the same β can occupy different states and cell types, with their future shaped by their past trajectories: their fate is path dependent.

Once major cell types have been reached, cells navigate into increasingly narrow canyons, each representing distinct states within a type. These divergences do not conclude with the establishment of cell types and young, healthy, i.e. optimal states. Instead, we propose that this process continues as Cell Potency further declines with aging, leading to the formation of suboptimal minima in which cells can get trapped. In the ageing regime, numerous local minima emerge, analogous to the progressively complex branching observed in river deltas.

We attribute these suboptimal local minima to age-associated diminished function. They arise from the gradual deterioration of cellular molecular composition over time resulting from imperfect repair mechanisms, which evolved mainly to preserve fitness until successful reproduction.^10^ Beyond that, damages can compound and eventually impair function giving rise to suboptimal but stable local minima. These manifest as the sum of different defects in different cells and collectively give rise to stereotypical macroscopic aging phenotypes, such as the appearance of wrinkles in aged skin or, for instance, sarcopenia.

Our model operates on two time scales: 1) a slow descent along the Cell Potency β-axis, which naturally occurs over extended periods of time such as development and eventually aging and 2) a rapid movement along the Cell State axis for each given potency β, representing the swift attainment of a minimum driven by the Cell Potential. Young cells at high potency can readily recover from stress by returning to optimal basins, they are resilient. In contrast, aged cells with low potency are often trapped in suboptimal local minima and therefore functionally impaired with an unhealthy future trajectory. A trapped cell is fragile and through continued stress deteriorates further over time; it becomes increasingly unlikely to return to the cell type optimum. Its diminished capacity for intrinsic rejuvenation manifests itself as ageing but, in our model, the information of a youthful state is not lost.

The Cell State Landscape is consistent with age-associated increasing molecular noise, age-associated cell state “drifting” and also with transient, small scale dedifferentiation, i.e. local potency increase, as it can occur in response to stress and injury.^11–13^ Additionally, since we think of the Cell State Landscape as species-specific, the varying onset of aging phenotypes across the tree of life can be readily explained by different rates of decline of β.

## Cell Annealing — a phenomenological model of rejuvenation

How can cells escape local cell state minima and regain youthful, healthy optima? Our model proposes that the potency β determines the regions of the Cell State Landscape accessible to a cell. We define this as the cell’s ‘Möglichkeitsraum’: the portion of the hyperdimensional manifold accessible by a given cell at a given time. This space is small for cells at low potency as only minima within the same cell type valley are easily accessible. In contrast, at high potency, qualitative transformation is possible with access to entirely different cell type valleys. In this view the generation of induced pluripotent stem cells (iPSCs) is an expansion of the Möglichkeitsraum through increase of Cell Potency to a level that ultimately allows access to all cell types and states.^14^

Multiple studies have shown that time-restricted expression of four transcription factors (4F) can reverse age-associated phenotypes both in vitro and in vivo, and extend lifespan as initially demonstrated in a mouse progeroid model.^15^ This partial reprogramming and derivative protocols have been shown to be effective across cell lineages, tissues, and organs.^16–18^

Strikingly, cellular rejuvenation has also been achieved using 4F-independent approaches including through manipulation of ECM- or systemic signalling, employing chemical cocktails that alter the epigenome and changes to the metabolome.^19–26^ Despite their diversity, these interventions converge on overlapping, similar outcomes, suggesting that there could be a broader underlying principle that transcends the specific molecular pathways involved. These interventions appear to not simply overwrite cell identity; rather, they seem to alter cells such that their systems can self-correct. In our model this can be explained by a process we term Cell Annealing.

In materials science annealing restores old, brittle metals by heating them to above the recrystallization point followed by a slow cooldown.^27^ This relieves internal stresses and restores structural order, recovering macroscopic properties like malleability and toughness. Thermodynamically, annealing briefly increases entropy, allowing the system to explore new configurations and settle into optimal states, guided by the free energy landscape governing crystal formation.

We propose that aged, unhealthy cells can anneal in a similar manner: a moderate, transient increase, a mild shock, to Potency β allows the cell to escape local minima, briefly extend their Möglichkeitsraum and rapidly re-anneal to the optimal state at this elevated potency. Upon subsequent reduction of β, the cell remains in or close to its optimal Cell State position (Fig. 1c). We propose that this process does not necessitate dedifferentiation.

Reprogramming effectiveness is age-dependent and, in our model, shaped by a cell’s initial state and the dynamics of potency modulation. It can fail if cells are trapped too deeply in troughs and conversely, sustained, strong β-elevation can lead to identity loss or even iPSC formation.^28^ In contrast, mild and transient β elevation could, in principle, drive controlled annealing to optimal states without loss of cell identity. This may explain both the broad capacity for partial reprogramming across cell types and the observed cell-by-cell heterogeneity, which likely reflect different cell states before treatment. Our model may also explain transcription factor-independent rejuvenation protocols: as long as an intervention rapidly elevates Cell Potency to the right level and for the right duration, the cell’s intrinsic capability to reconfigure to a youthful state is unlocked. This can happen without explicit instructions for an embryonic or other natural gene expression program and does not require external information transfer towards a healthy target state. In Cell Annealing, instead of postulating an error-free back-up copy of youthful information, that information is stored in a distributed fashion across modalities.^29^

## Discussion and future directions

If cellular age reversal requires externally guided changes to cell trajectories, a conventionally designed intervention will require: (1) a comprehensive understanding of a healthy cell state of a given type to establish a target state, and (2) a means of conveying that target information to the cell, effectively enabling a controlled shift from an old, unhealthy to a youthful trajectory. The emerging, dauntingly complex network of age-associated molecular phenotypes makes this appear unrealistic. Nevertheless, cellular rejuvenation protocols have shown positive effects across remarkably different types of cells and types of interventions, suggesting that instead there may be a universal approach to reverse age-related phenotypes. Cell Annealing offers a simple, unifying, framework to model these processes in analogy to restoration of crystal structures of metals by transient heating. Annealing is a remarkably universal phenomenon with applications ranging from numerical simulation to models of trauma dissolution.^30^ We propose to apply it to cell states.

It will be important to establish empirical measures as proxies for Cell Potency. For instance, the broad permissive transcription seen in stem cells suggest that transcriptome breadth or age-associated transcriptomic variability could be a candidate measure. Also, epigenetic features like DNA methylation and chromatin patterns or pattern distributions across cell populations, as well as a cell’s capacity to transdifferentiate to a new cell type can be considered. Advances in multimodal single-cell technology will allow to refine and test these ideas.

Many important biological aspects, such as cell population dynamics or information transfer from single cells to tissues and organs are currently beyond the scope of this model. However, we hope this early sketch of Cell Annealing can provide a conceptual scaffold and a starting point for the development of an explanatory framework in the quest for therapeutic strategies for reversing cellular and, ultimately, organismal aging and disease.
